# The Role of Problem-Based Learning in Preparing Medical Students to Work As Community Service-Oriented Primary Care Physicians: A Systematic Literature Review

**DOI:** 10.7759/cureus.46074

**Published:** 2023-09-27

**Authors:** Heather M Forbes, Munir S Syed, Octavia L Flanagan

**Affiliations:** 1 Medical Education, Lake Erie College of Osteopathic Medicine, Horseheads, USA; 2 Pathology, Histology, Lake Erie College of Osteopathic Medicine, Elmira, USA; 3 College of Osteopathic Medicine, Lake Erie College of Osteopathic Medicine, Elmira, USA

**Keywords:** disease prevention and control, clinical competence, interpersonal skills, board exam scores, primary care, medical education, pbl curriculum

## Abstract

The number of primary care physicians in the United States is dwindling rapidly, and osteopathic medical schools are embracing the challenge of leading students toward a career in primary care to meet this need. In recent years, the Problem-Based Learning (PBL) curriculum in medical education has emerged as a patient-centered, social-justice-focused methodology. The unique format of PBL centered around patient cases allows learning through community-based medicine, promoting medical graduates’ entry into primary care. Through exploring the literature on this topic, the research question posed for this review is as follows: How have the skills gained in PBL been effectively preparing medical students to become community service-oriented primary care physicians, and how can we qualitatively and quantitatively assess a learner’s preparedness to engage in primary care work?

The variables studied were board licensing examination scores, clinical competence, and interpersonal skills, all of which emerged as common ways to assess learners’ preparedness to work in primary care. The methodology of this literature review was organized using a Preferred Reporting Items for Systematic Reviews and Meta-Analyses flowchart to describe how articles were selected and synthesized to evaluate the variables. The results revealed the variables to be consistent strengths of PBL students, particularly clinical competence, and interpersonal skills, both of which are key in working in primary care and any clinical specialty. Since early in its implementation, literature has demonstrated the tendencies of PBL students to be interested in and later work in primary care, though little follow-up has been done recently. The question of why this phenomenon exists was largely answered by our literature review.

In conclusion, through our analysis of the existing literature, the authors demonstrated that the PBL curriculum helps foster students’ desire to serve patients. Limitations of the literature included small sample sizes, heterogeneous analysis methods, limited inclusion of qualitative assessment of student progress, and limited existing data on the prevalence of PBL in medical schools, as well as the entrance of PBL graduates into primary care careers.

## Introduction and background

The role of community health in primary care

It has become evident in recent years that investment in community health through preventive primary care is essential in the United States [1]. In this paper, primary care physicians are care providers in the fields of family medicine, pediatrics, obstetrics and gynecology, and internal medicine. It is projected that between 2019 and 2034, there will be a shortage of between 17,800 and 48,000 primary care physicians [2]. In response to this increasing demand for primary care physicians in the United States and other countries, organizations are initiating programs that encourage medical students to enter a primary care training path [3]. At the same time, medical schools and residency programs are evolving to focus on developing their students’ skills in community health to better serve underserved populations and improve healthcare access through primary care [1].

The literature suggests that primary care provides patients with a longstanding relationship with their physician, in which trust can be built, and the concept of disease prevention is the focus of the care they receive [4]. However, most primary care physicians are located in hospitals, leading to inaccessible care for many uninsured people who are unable to get appointments [5]. Programs focused on community empowerment, health promotion, and disease prevention located in impoverished areas of our country have been shown to be very effective if primary care services are provided with little to no payment from the patient required [4].

In the late 1960s through the early 1970s, Tufts Medical School built such a program within a Federally Qualified Health Center (The Tufts Delta Health Center (TDHC)) for the community of impoverished people of color residing in the Mississippi Delta employed as sharecroppers without health insurance benefits [6]. This Center allowed medical students from Tufts to care for patients within this clinical setting, which focused on preventative, community-based medicine. As the TDHC was a clinical arm of the Tufts School of Medicine, it provided an additional opportunity for students to experience a community health focus with a population of uninsured patients whom these students would not find within medical practices and hospital clinics. The framework of TDHC focused on empowering the citizens of the community to create elder support and supplemental food programs with the aid of Federal Grant monies, with a community focus on tearing down the systemic barriers that face persons of color living in poverty in the United States [6].

Problem-Based Learning and community health

Tufts Medical School, and many other medical schools in subsequent years, used medical education to bridge the gap between primary care and community health. Problem-Based Learning (PBL) curriculum, adopted by many medical schools through recent decades, is another initiative bridging this gap. PBL stands in a convenient place to make an impact in the community, as it is centered on developing traits of primary care physicians, promoting the prevention of disease, and an ongoing relationship that fosters trust with people that have traditionally been ignored by medicine.

The PBL curriculum is a student-centered instructional approach focused on a team approach to medical decisions using case studies [7]. Osteopathic education, a holistic approach to care with a focus on disease prevention, is emphasized in the PBL cases that students work through. This is presented in cases with a focus on family and community and psychosocial aspects of holistic care and the implication of not only disease prevention but also the focus on primary care with an ongoing relationship between patient, family, and physician.

PBL was initially established by disgruntled physicians who wanted to make medical education more engaging [8]. PBL can be characterized by (i) learning in small groups; (ii) a medical educator facilitating learning in the group rather than acting in the “expert” role; and (iii) learning by using problems/cases that direct discussion among group members as the case unfolds. (iv) Following class, students are encouraged to study on their own the case learning objectives that they need to develop either new knowledge or emerging concepts that need to be clarified [7].

The unique format of PBL leaves room for focus in discussion on social justice and community-oriented aspects of patient care. Models of community service through PBL have been shown to directly benefit local community partners such as private clinics [9].

The push from PBL to primary care

The PBL method of medical education has been shown to be effective in preparing students for a career in primary care incorporating clinical exposure to family medicine early in the curriculum [3]. In a 2017 study led by Tsigarides et al. that was conducted to determine if medical students in the PBL curriculum were more or less likely to choose a career in primary care, several papers found a significant difference between students taking the PBL track preferring a primary care focus at a clinically significant p-value of 0.05 more than other students in other medical teaching modalities outside of PBL [3]. Most notably, a paper by Peters et al. found that at the Harvard Medical School “New Pathway” PBL program, 40% of PBL students vs. 18% of traditional track students ultimately practiced primary care or psychiatry, rating their preparation to practice “humanistic medicine as higher” than their peers [10].

Results from studies conducted in the 1990s showed that five years after graduation, a slightly higher percentage of PBL students than non-PBL students had entered family practice (28% vs. 20%) and pediatrics (12% vs. 10%) [11]. More PBL students at the University of New Mexico School of Medicine were found to be serving in medically underserved areas (five times as likely), in public clinics, and as primary care physicians than non-PBL students (49% vs. 44%) [12]. Since then, research has shown that efforts to provide access to comprehensive preventive screenings and treatment must be prioritized to reduce the inaccessibility of medical care for underserved populations [4]. Providing access to comprehensive preventive screenings and treatment can play a role in reducing and eliminating at least some racial inequities in health. The early transition of PBL programs to being community-oriented has also been associated with improvements in physicians’ practice of preventive care and continuity of care, as measured by community mammography screening rates and disease-specific prescribing rates [13].

Through encouraging students to practice preventive care and continuity of care in primary care fields, the PBL curriculum is developing effective primary care physicians. This is evidenced by the fact that as of 2003, 70% of the medical schools in the United States accredited by the Liaison Committee on Medical Education, which is a quality assurance program that determines whether medical education programs meet established standards, use at least some degree of PBL in their preclinical years [14]. Since the early 2000s, the popularity of the PBL curriculum has grown significantly, spreading to some 500 higher learning institutions [8]. There is no recent research on the efficacy of community-oriented PBL programs on local patient populations. Furthermore, little to no literature exists analyzing the presence of PBL curriculum-educated physicians working in primary care in communities of high need. Given the supportive literature that suggests that a PBL curriculum encourages medical students to pursue a community-based primary care medical focus, the authors of this paper are interested in exploring what makes a student who follows a PBL curriculum become more interested in becoming a community service-oriented primary care physician.

This paper considers the important role of the PBL curriculum in medical education in filling the need for primary care physicians who are community-oriented in their commitment to caring for the whole patient. For our purposes, we define a community service-oriented primary care physician as one who prioritizes treating all patients with longitudinal care that is responsive to their community’s needs and is rooted in trust and preventive care. Community health, namely addressing the social and health needs of any diverse group of people, is central to the scope of practice of any community service-oriented physician.

Using a systematic literature review to investigate this outcome that has been scarcely considered in the medical education literature in the last 18 years, the research question of interest that developed was, how have the skills gained in PBL been effectively preparing medical students to become community service-oriented primary care physicians? A further question that emerged through our research was, how can we qualitatively and quantitatively assess a learner’s preparedness to engage in primary care work? The aim of this study is to explore whether the PBL method of learning encourages medical students to consider becoming community service-oriented primary care physicians and if this method of teaching has additionally improved scores on national board certification exams (including both United States Medical Licensing Exam (USMLE) and Comprehensive Osteopathic Medical Licensing Examination (COMLEX)), along with clinical competence and interpersonal skills.

The three variables included in our study were USMLE/COMLEX performance, clinical competence, and interpersonal skills. These are all valid measures of medical student performance, present throughout the selected articles, and have been deemed as important in career work as a community-oriented primary care physician. These three measures of a medical student’s achievement are used in the literature as a means of comparing the effectiveness of the PBL curriculum against non-PBL curricula. From our research, these markers seem to stand out as competencies that PBL students excel at, and, subsequently, prepare them effectively to work as engaged, community-oriented primary care physicians. Our findings will be useful for both students considering whether they should engage with the PBL curriculum during medical school, as well as schools, educators, and governing bodies, when assessing the effectiveness of PBL and whether it should be implemented in their programs.

USMLE/COMLEX scores

To graduate medical school, students must successfully complete the requirements of medical school. Required board tests include either the USMLE Step 1 and 2 and/or COMLEX Level 1 and 2. USMLE Step 1 is a basic science exam conducted by the National Board of Medical Examiners (NBME), traditionally taken following the first two pre-clinical years of medical school. Following this, Step 2, testing clinical knowledge, is usually taken after the third year of medical school. In parallel to this are the National Board of Osteopathic Medical Examiners’ (NBOME) COMLEX Level 1 and Level 2, taken by osteopathic medical students, both established as ways of measuring knowledge in the basic sciences [15]. This was our first measure.

Clinical competence

Another marker of medical student development is the clinical skills that they demonstrate in clinical rotations; this is a marker of the self-efficacy they have achieved throughout their training. A common assessment tool used is the Chart Stimulated Recall and Assessment of Clinical Reasoning in the Workplace, which, with the help of faculty assessors, leads students to measure their medical knowledge, patient care, and procedural skills development [16]. Skills assessed in this domain range from confidence, self-directed learning, self-awareness, diagnostic skills, and clinical reasoning. Along with methods of student self-assessment through similar surveys, clinical reasoning can be assessed; this was our second measure [16].

Interpersonal skills

Along with confidence in clinical skills comes the ability to work well with others, from colleagues to family members and patients. Interpersonal skills are one of the PBL student’s strongest skills [7]. A valid way to assess the development of interpersonal skills and professionalism built through the PBL curriculum is through faculty assessment of students. A common method used to assess this is Multisource Feedback (MSF), which involves multiple surveys that are completed by different people who interact with the learner [16]. This and other checklist evaluations are appropriate for competencies that can be broken down into actions [17]. Data from MSF can be used to make judgments on the interpersonal and communication skills of a learner; this comprised our third measure [16].

## Review

Methodology

**Figure 1:**
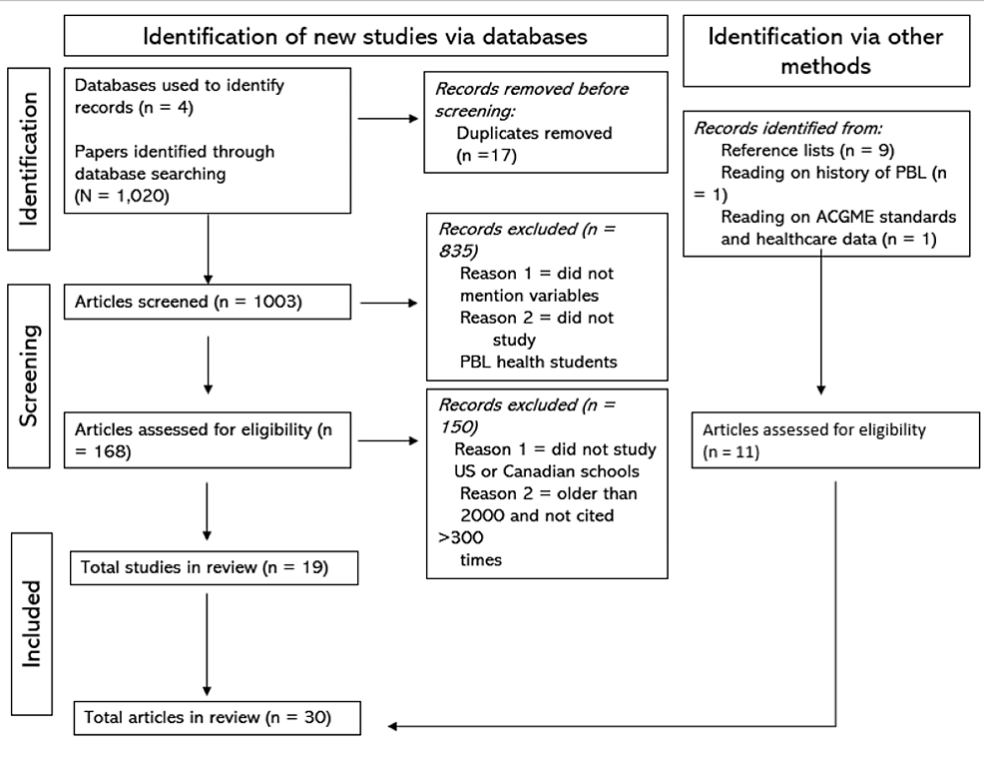
PRISMA flowchart of the systematic literature review. N: number of articles selected; ACGME: Accreditation Council for Graduate Medical Education; PRISMA: Preferred Reporting Items for Systematic Reviews and Meta-Analyses

To organize the results of the study the authors used PubMed, EBSCO, Taylor & Francis Online, and Science Direct to locate sources, relying on others within these sources when a particular article was not accessible. On PubMed, we searched “PBL residency performance,” “PBL + primary care,” and “community health + underserved.” On EBSCO, we searched “PBL + interpersonal skills + medical students” and “PBL + test scores + medical students.” On Taylor and Francis Online, we searched “PBL method + residency outcome.” On Science Direct, we searched “PBL + ethics + medical school” and “PBL + primary care + medical practice.” We narrowed searches down to articles published in the United States and Canada to limit this review to programs that occur following undergraduate studies. This yielded 1,020 articles. This process is detailed below in Figure 1.

Literature Review Design

Within the 1,020 articles, the authors manually sorted through titles to exclude irrelevant ones. In total, 17 duplicates were removed, leaving 1,003 articles. From this, further filtering indicated that 835 articles either did not mention the exact variables in question or did not study PBL students in health fields. Once we had our valid measures in mind, we filtered through 168 articles, from which we chose only articles that explicitly mentioned board/exam scores, self-assessment of clinical competence, or interpersonal skills as assessed by professors or evaluators. Through this exclusion criteria, we further excluded articles that did not compare PBL students to either national averages or non-PBL curriculum peers. Additionally, we analyzed papers older than 2000 to find landmark articles. As a criterion, we only used those articles older than 2000 that were cited by at least 300 other papers. This left us with the 18 articles relevant to our research. Using these, we also found 12 articles that provided relevant background and context about PBL, guiding our research into primary care education and community involvement as physicians.

We critically analyzed each article to note its sample size, methodology, outcome measures, and results. Studies with both qualitative and quantitative findings are included and will be presented to demonstrate the multifaceted aspects of medical education. We noted whether each article spoke to PBL students’ (1) success on either USMLE Step 1/COMLEX Level 1 and/or USMLE Step 2/COMLEX Level 2, (2) clinical skills, or (3) interpersonal skills.

Only one paper included was primarily qualitative (Wormley et al.) while the rest were primarily quantitative [18]. This is perhaps due to the data-driven nature of implementing new educational methodologies. Of the papers included, the mean sample size was 150.05. The papers included ranged from the years 1993 to 2020 (27 years). It is important to note that while most group comparisons were relatively balanced, Richards et al. looked at 88 PBL and 364 non-PBL students, which may have led to their data being slightly skewed [19]. All but three articles reviewed PBL programs at medical schools, while one looked at a physical therapy program, one looked at a dental school, and one looked at a Master of Public Health (MPH) program. Albanese and Mitchell never explicitly said how many sources were in their review [20].

Results

Measurement Tools in Articles

Outcome measures were assessed using tools ranging from surveys and questionnaires to retrospective data analysis, to literature review and analysis, to pure analysis of test score data. Questionnaires and surveys were largely focused on students’ perspectives on the PBL curriculum and their own experiences and strengths following engagement with it.

All included studies directly compared outcomes between PBL curriculum students and non-PBL curriculum students. Overall, seven of 18 papers looked at the board scores of PBL students as an outcome (either USMLE or COMLEX). Further, eight of 18 papers looked at the clinical skills of students as measured by their self-assessments, and one of these eight looked at supervisor ratings of clinical abilities. Five of 18 papers looked at interpersonal skills as measured by evaluator assessments of students, two looked at interpersonal skills as measured by student self-assessments and one of these eight looked at interpersonal skills as measured through provider (faculty and resident) self-assessment. Three of 18 papers looked at more than one outcome measure.

Of the 18 papers included, four were literature reviews and 14 were studies that looked at either retrospective data or current data regarding individual students, classes, or schools. All except one compared PBL students to non-PBL students. Two articles were cohort studies and five articles were cross-sectional; the rest were retrospective or comparing historical data. Due to the heterogeneity of studies included, each study employed a slightly different method.

Study Findings in Articles

USMLE/COMLEX scores:Early reviews of literature did not show data in favor of PBL. By looking at basic science examination performance data available at the time, Albanese and Mitchell concluded that in six of the 10 studies included, the overall basic science scores of students in “conventional curricula” were higher than their PBL counterparts [20]. This was measured in most studies by NBME Part 1 Examination, synonymous with USMLE Step 1. These lower scores are in contrast with the on average higher scores in the clinical part of the NBME (Step 2), signifying PBL’s success in promoting practical skills [20].

In the same year, Vernon and Blake conducted a meta-analysis of the literature regarding PBL’s effect on NBME Step 1 scores [21]. Looking at eight reports, they concluded that the measures for the NBME Step 1 were found to be “significantly heterogenous” between standard and PBL curricula, more than would be expected from chance [21].

Despite these initial results, following years of refinement, PBL led to upward trends in student USMLE scores. A study conducted at the University of Missouri-Columbia (UMC) showed that before their implementation of the PBL curriculum in 1996, students were on average scoring at or below the national mean on USMLE Step 1, the mean scores for the four classes of their new PBL curriculum were on average as much as eight points above the national average [22]. Of the four class years studied, the three later class years (1997-1999) were also above the national mean on USMLE Step 2 [22].

Ten years later, Hoffman et al. again looked at medical students at UMC before and after the implementation of PBL comparing their USMLE Step 1 and Step 2 scores to national averages [15]. They found that mean scores of six of 10 comparisons for USMLE Step 1 and six of nine comparisons for USMLE Step 2 were significantly higher for UMC PBL students (p < 0.01) than for first-time examinees nationally [15].

Despite those promising findings, Eniarson and Cariaga-Lo looked at 689 students who took USMLE Step 1 and 540 students who took USMLE Step 2 over a seven-year span at Wake Forest University School of Medicine, only to find a lack of statistically significant difference between PBL and standard curriculum students [23]. Still, this provided evidence that the PBL curriculum continued to promote the acquisition of basic science knowledge.

In 2009, Thomas et al. found that current obstetrics and gynecology residents who were PBL students performed significantly better on USMLE Step 2 than their counterparts from traditional curriculums. This is further evidence that the improved scores of PBL students continue into the more clinically based Step 2 exam [24].

Most recently, Zaveri et al. showed that Lake Erie College of Osteopathic Medicine (LECOM) Bradenton outperformed the national COMLEX Level 1 score every year except one [25]. Following the implementation of NBME Comprehensive Basic Science exams to assess learning and retention, they found that the school’s mean COMLEX Level 1 score has been between 26 and 55 points above the national mean score for 12 of 13 years studied [25].

Clinical competence: As early as 1993, Vernon and Blake found that in all seven studies reviewed regarding clinical competency in PBL, ratings by faculty supervisors were either more positive for students in PBL or not significantly different than the conventional curriculum. The trends they found in all aspects of rating the clinical performance of PBL students showed significantly higher ratings for PBL students [21].

Primary Care Curriculum (PCC) graduates University of New Mexico School of Medicine’s PBL track felt better prepared in their teamwork, clinical reasoning, diagnostic skills, and physician-patient relationships than their traditional track peers, as measured by self-evaluations, rating themselves higher than their traditional counterparts in 11 of 17 categories in both cognitive and non-cognitive areas (ranging from clinical reasoning, coping with uncertainty, and diagnostic skills to preventive care issues) [12].

In the same year, Fields et al. looked at the practice characteristics of PBL-educated physicians to consider clinical competence [11]. Five years after graduation, a slightly greater percentage of PBL students worked in family practice (28% vs. 20%) when compared to non-PBL peers [11].

Through a review of the literature regarding physician competency both through self-reflection and observer assessment, Koh et al. found that based on the observed assessments, seven competencies, such as diagnostic skills, coping with uncertainty, and responsibility and peer-appraisal, were stronger in PBL students than their traditional counterpart peers [26].

Distlehorst et al. looked at self-evaluations of standard curriculum versus PBL curriculum-educated residents [27]. Most relevant was that in the third postgraduate year, standard graduates did not rate themselves higher than any PBL peer on any clinical skill; however, PBL graduates rated themselves higher on self-directed learning habits (0.82) [27]. This is evidence that graduates of both tracks feel equally well-equipped for clinical practice [27].

Hou found that the PBL curriculum allowed students who might traditionally rank lower to gain more confidence in their ability to problem-solve and actively contribute in the clinical setting [9]. Through having students reflect on their experiences working through PBL on community health projects, he learned that students valued the active learning, discussions, and teaching experiences gained through the clinical work portion of their experience [9].

Tsigarides et al. looked at whether the PBL curriculum made students more likely to enter primary care. Based on student self-reflection present in many papers, there was no significant difference in career choices in seven out of 11 papers [3]. Despite this finding, the paper comments that many factors outside of the school curriculum likely contributed to a PBL student’s decision to enter primary care, including their confidence in their clinical skills [3].

According to a study by Margolius et al., clinical skills learned in PBL that students rated most highly as “helpful during core clinical rotations include comfort discussing concepts, identifying key information, presentation skills, interpersonal skills, diagnostic thinking, finding information, self-awareness, and organizing information” [28]. These skills are taught early on in PBL and correlate to improved patient care once in the hospital. These skills are key to enabling community-focused care, whether through outpatient preventative care clinics or through reaching underserved communities.

Interpersonal skills:Albanese and Mitchell found that studies based on separate graduates from a PBL and conventional curriculum school showed that PBL graduates view their preparation in humanistic areas and preventative care more positively than traditional curriculum peers, demonstrating how the curriculum promotes interpersonal skills [20].

Richards et al. found that PBL students outperform traditional curriculum students in areas of self-realization and achievement via independence in third-year clerkships, indicative of their interpersonal skills promoted throughout the PBL pre-clinical curriculum [19].

In PBL, students are encouraged to attend to collaboration processes through their reflection and through the interdependence of learning, but they do not necessarily know how to deal with the collaborative aspects of discussion effectively [29]. Through clinical experiences, teamwork skills became apparent to PBL students: they excel at communicating with co-workers, patients, and families, as well as conveying information, all valuable team-based interpersonal skills [18].

Looking specifically at the Harvard New Pathway (PBL) on humanistic knowledge, attitude, and skills, Vernon and Blake found that the PBL students scored higher on five skills related to communication and data collection, indicating the effectiveness of this curricular change in orienting students toward patient care, particularly building interpersonal skills [21].

By looking at preventative care outcomes that were accessible in a database of graduates, Tamblyn et al. found that for Sherbrooke University in Quebec, transitioning to a community-oriented PBL curriculum was associated with “substantial and statistically significant improvements in preventive care and continuity of care,” markers of effective implementation of interpersonal skills measured by patient outcomes [13].

In 2007, Thammasitboon et al. compared responses of Harvard School of Dental Medicine (HSDM) PBL students to non-HSDM students using 12 competencies in communication, general dental knowledge, and pre-clinical skills, adapted from ACGME standards. Using Mann-Whitney tests comparing self-assessments of PBL and non-PBL dental students showed significant differences (p < 0.05) for almost all competencies expected to be enhanced by the PBL method including ability in communication with patients, critical thinking, independent learning, self-assessment, teamwork, and performance in small group settings, all necessary clinical skills [30].

Distlehorst et al. compared the self-ratings of PBL and non-PBL-educated residents throughout their years of residency [26]. At the end of the postgraduate year, there were two areas in which the self-ratings of PBL graduates were higher than those given by supervisors, one particularly relevant to interpersonal skills: “communication skills” [27].

Lochner et al. found through provider self-assessment that when the University of Wisconsin Madison’s Family Medicine Residency program was reworked to include more curriculum about community health, namely, through clinic-based population modules and community-engaged partnerships, both faculty and residents felt more able to intuitively use their interpersonal skills and serve patients in their work as primary care physicians [3].

Students engaged in a Physical Therapy (PT) curriculum reported that their learning method provided them with many opportunities to build confidence and showcase independent rationale for clinical decision-making during lab practicals [18]. These are skills that translate well to working independently as a doctor with patients in need. Furthermore, these are essential skills in any specialty, particularly primary care in which many co-existing disease processes and risk factors might be at play. Table 1 presents the entirety of the results of this study.

**Table 1:** Findings of the literature review. Systematic research of the literature led to the inclusion of 19 relevant studies to address the research question outlined in the first section of the review paper. PBL: Problem-Based Learning; STROBE: Strengthening the Reporting of Observational Studies in Epidemiology; CONSORT: Consolidated Standards of Reporting Trials; USMLE: United States Medical Licensing Examination; ANCOVA: analysis of covariance; COMLEX: Comprehensive Osteopathic Medical Licensure Examination

Year	Authors	Article title	Outcome measured	Subjects	Methodology	Findings/Conclusion
2017	Lochner et al. [1]	Transforming a family medicine residency into a community-oriented learning environment	Interpersonal Skills	125–142 faculty and 42 residents	Kruskal-Wallis and chi-square tests to compare survey responses	Community health integration into primary care residency leads to more confident, capable, and involved residents
2017	Tsigarides et al. [3]	Does a PBL-based medical curriculum predispose training in specific career paths? A systematic review of the literature	Clinical skills	11 studies	STROBE and CONSORT quality assessment checklists to analyze observational studies and randomized control studies, respectively	Seven of 11 studies found no difference in specialty choice between the PBL and standard curriculum. Three studies showed an increased number of PBL graduates pursuing primary care
2014	Hou [9]	Integrating problem-based learning with community-engaged learning in teaching program development and implementation	Clinical skills	162 Master of Public Health (MPH) students	Five-point Likert scale regarding PBL community-based experience	Students reported positive impacts on their learning, critical thinking skills, as well as collaboration with peers. The survey also showed that PBL increased student self-confidence and self-awareness
1996	Fields et al. [11]	PBL and primary care career choice: a complex relationship	Clinical skills	Eight medical schools	Analysis of phone interviews focusing on the characteristics of medical school, primary care opportunities, and career choice, role of PBL	Comparing tracks, in one school, five years after graduation, a slightly higher percentage of PBL students had entered family practice (28 vs. 20) and pediatrics (12% vs. 10%)
1996	Mennin et al. [12]	A survey of graduates in practice from the University of New Mexico’s conventional and community-oriented, problem-based tracks	Clinical skills	33 graduates from the PBL track	A survey administered and analyzed using two-way analyses of variance, logistic regression, and chi-square	PBL students felt more prepared in their teamwork and clinical reasoning skills and went on to serve in medically underserved areas. PBL students also volunteered more
2005	Tamblyn et al. [13]	Effect of a community oriented problem based learning curriculum on quality of primary care delivered by graduates: historical cohort comparison study	Interpersonal skills (measured by performance in preventive care)	751 doctors at four medical schools	Linear regression analysis using data from service claims	PBL learning leads to increased preventative, patient-focused primary care
2006	Hoffman et al. [15]	Problem-based learning outcomes: ten years of experience at the University of Missouri-Columbia School of Medicine	Board scores (USMLE Step 1)	19 PBL classes	Statistical analysis	Curricular changes including PBL led to improved student performances on the national licensing examinations that have persisted over a decade
2018	Wormley et al. [18]	Students’ perspectives of core value development in a modified problem-based learning program	Interpersonal skills	27 students from PBL Physical Therapy program	Analyzed transcripts of interviews for key phrases; themes found related to core values	Teamwork skills became apparent to the students during their clinical affiliations: they excelled at communication with their co-workers, patients/families, and effectively conveying information
1996	Richards et al. [19]	Ratings of students’ performances in a third-year internal medicine clerkship: a comparison between problem-based and lecture-based curricula	Interpersonal skills	88 PBL and 364 non-PBL students	ANCOVA analysis; four scales	PBL curriculum is associated with higher medicine clerkship performance ratings as measured by internality, norm-favoring, self-realization, and achievement via independence
1993	Albanese and Mitchell [20]	Problem-based learning: a review of literature on its outcomes and implementation issues	Board scores, interpersonal skills	English PBL literature from 1972 to 1992	Meta analysis of literature - effect size and p-values	PBL scores on USMLE comparable to standard curriculum. However, better interpersonal skills of PBL students
1993	Vernon and Blake [21]	Does problem-based learning work - a meta-analysis of evaluative research?	Board scores, clinical skills, interpersonal skills	22 studies	Statistical analysis; effect size of studies, denoted by d	Supports higher clinical functioning and academic process in PBL students
2000	Blake et al. [22]	Student performances on Step 1 and Step 2 of the United States Medical Licensing Examination following implementation of a problem-based learning curriculum	Board scores (USMLE Step 1 and USMLE Step 2)	Six classes (four of PBL and two non-PBL) of medical students	Multivariable analysis and comparison to nationwide data	Improved USMLE Step 1 and Step 2 scores for PBL students following major curriculum change (above the national average)
2008	Eniarson and Cariaga-Lo [23]	Influence of curriculum type on student performance in the United States Medical Licensing Examination Step 1 and Step 2 exams: problem-based learning vs. lecture-based curriculum	Board scores (USMLE Step 1 and USMLE Step 2)	689 students	t-test analyses between students taking Step 1 and Step 2 in PBL vs, traditional track over a seven-year period	Statistically significant main effects noted by cohort year and curricular track for both the Step 1 and Step 2 examinations
2009	Thomas et al. [24]	Problem based learning and academic performance in residency	Board scores (USMLE Step 2)	35 residents	t-test analysis	PBL students did better on USMLE Step 2 than their traditional track peers
2019	Zaveri et al. [25]	Changes to an active learning curriculum in osteopathic medical education: effects on exam outcomes and board scores	Board scores (COMLEX Level 1 and COMLEX Level 2)	145–195 students in 12 class years	t-tests and Fisher’s exact tests; statistical analysis with Rv3.5.1	PBL method outperforms national COMLEX Level 1 average in every year but one. Mean Level 1 score has been 26–55 points above national average in every year studied
2008	Koh et al. [26]	The effects of problem-based learning during medical school on physician competency: a systematic review	Clinical skills	13 studies	Systematic analysis of 37 competencies in literature review	Improved social and cognitive dimensions after PBL method
2009	Distlehorst et al. [27]	Supervisor and self-ratings of graduates from a medical school with a problem-based learning and standard curriculum track	Clinical skills and interpersonal skills	Performance ratings of 453 residents	Fisher’s exact t-test analysis of two tracks	Graduates from the PBL track gave higher ratings to themselves than graduates from the standard track on self-directed learning habits (0.82)
2020	Margolius et al. [28]	Students perceive skills learned in pre-clerkship PBL valuable in core clinical rotations	Clinical skills	35 PBL students	Analysis of returned survey results	Skills learned in PBL that were useful: comfort discussing concepts, identifying key information, presentation skills, interpersonal skills, diagnostic thinking, self-awareness
2007	Thammastiboon et al. [30]	Problem-based learning at the Harvard School of Dental Medicine (HSDM): self-assessment of performance in postdoctoral training	Interpersonal skills	80 dental students (42 PBL)	Mann-Whitney test with significance of p < 0.05. Wilcoxon-signed ranks test	HSDM graduates rated themselves more highly than non-HSDM graduates on all competencies regarding interpersonal skills

Discussion

The authors analyzed literature review papers that explored the knowledge and skill development of PBL students that prepare them to work as community-oriented primary care physicians. The different papers included the effects of the PBL curriculum on medical students’ board exam (USMLE/COMLEX) scores, clinical competence, and interpersonal skills. This review concluded that PBL students score the same, if not better, than non-PBL students, on board exams. PBL students demonstrated better clinical competence and interpersonal skills when on rotations than their non-PBL peers.

Early on, Fields et al. found in their analysis of eight medical schools that provided data about their PBL graduates that most had a slightly higher percentage of PBL vs. non-PBL students beginning working in family practice or pediatrics, both primary care fields [11]. In all included schools, the difference between PBL and non-PBL students was about 10% more PBL graduates entering primary care [11]. They acknowledged, however, that their study would have benefitted from a five-year follow-up, as many students choose to further specialize [11]. More recently, Tsigarides et al. showed that early exposure to family medicine, among other factors, can lead to PBL graduate primary care [3]. However, they acknowledged that primary care may appeal to graduates with “different personality traits,” which might lead them to choose PBL [3]. As such, they argue that there are confounding factors beyond the PBL curriculum itself that contribute to students’ clinical competence.

USMLE/COMLEX Scores

Of the seven papers included that looked at board exam scores of PBL students, five found PBL students to score at or above the national average (or higher than their traditional curriculum peers). Four of the seven examined found that PBL students scored higher on USMLE or COMLEX 1, and those who did not tended to be older or based on a smaller sample size. All three papers that looked at USMLE Step 2 indicated statistically significant higher scores of PBL students [15,24,23]. This is a marker that students are well-equipped by their education to work as community service-oriented primary care physicians.

Clinical Competence

Of the eight papers looking at clinical competence, six had statistically significant results in favor of PBL students. Albanese and Mitchell noted that in their early years, PBL students had deficits in their ability to engage in “backward reasoning,” which led to some weaknesses in clinical performance [20]. More recent papers show trends in favor of PBL students’ skills in the clinical setting. Improved clinical skills domains include improved social and cognitive dimensions in the clinical setting [26], improved ability to use critical thinking in the clinical setting [12], improved diagnostic skills, and comfort in discussing difficult topics with patients [28].

Interpersonal Skills

All eight papers looking at PBL students’ interpersonal skills found statistically significant results favoring PBL students. As an exemplar of the improvement of interpersonal skills through experience in the community health setting, Tamblyn et al. found that at Sherbrooke University in Quebec, transitioning to a community-oriented PBL curriculum was associated with improved preventive care and other aspects that furthered students’ interpersonal skills [13].

USMLE scores, clinical competence, and interpersonal skills all contribute to graduates’ ability to work as community-oriented physicians. PBL students are achieving highly in these three areas, measured qualitatively and quantitatively. Much like PBL programs in recent years, successful international medical schools are being “more community-oriented and socially accountable” and, in so doing, are producing graduates that can “tend to the personal health care needs [of individuals] in the context of population health” [19]. It is evident that these needs continue to be areas for growth in medical education, and increasingly community health-oriented PBL programs are addressing these gaps. In so doing, they are producing physicians who can fill nationwide shortages, drawing on their strengths with their humanistic training through PBL.

## Conclusions

The variables studied are consistent strengths of PBL students. Though improvement of these variables over traditional curriculum peers took time, as the PBL curriculum became refined to suit its goals, so did the success of its students. Today, PBL students excel on board exams. Furthermore, their superior competency in the clinic, demonstrated both procedurally and through interpersonal interactions, is noted by evaluators and students themselves. Though this developed into a complex question because not enough literature exists explicitly on these topics, our literature review answered our initial question by showing that through bolstering students’ knowledge, confidence, and experience through clinical encounters, the PBL curriculum fosters students’ desire to serve patients through primary care. The development of the skills in question is key to students’ success as humanistic primary care physicians.

However, there were periods of time when the curriculum was not largely researched, despite its widespread implementation. Little investigation has been done recently to consider the engagement of PBL graduates in primary care careers. Similarly, no analysis has looked at the degree of PBL usage in medical schools today. Furthermore, the heterogeneous nature of PBL implementation across schools makes it difficult to study comprehensively. Now that more data on career outcomes exists, newer studies are needed to evaluate the current role of the PBL curriculum in producing primary care physicians on a large scale given the nation’s shortages. The sample size of some articles limits the power of their conclusions, as many are case studies on one or a few schools.
